# Mercury-Modulated Immune Responses in Arctic Barnacle Goslings (*Branta leucopsis*) upon a Viral-Like Immune Challenge

**DOI:** 10.1021/acs.est.2c07622

**Published:** 2023-03-20

**Authors:** Biyao Han, Hans van den Berg, Maarten J.J.E. Loonen, Rafael Mateo, Nico W. van den Brink

**Affiliations:** † Wageningen University, Division of Toxicology, Postal code 8000, NL-6700 EA Wageningen, The Netherlands; ‡ University of Groningen, Arctic Centre, Aweg 30, NL-9718 CW Groningen, The Netherlands; § Instituto de Investigación en Recursos Cinegéticos (IREC), Ronda de Toledo, 12, 13071 Ciudad Real, Spain

**Keywords:** mercury, immune toxicity, avian, barnacle goose (Branta leucopsis), arctic, exposure and effect

## Abstract

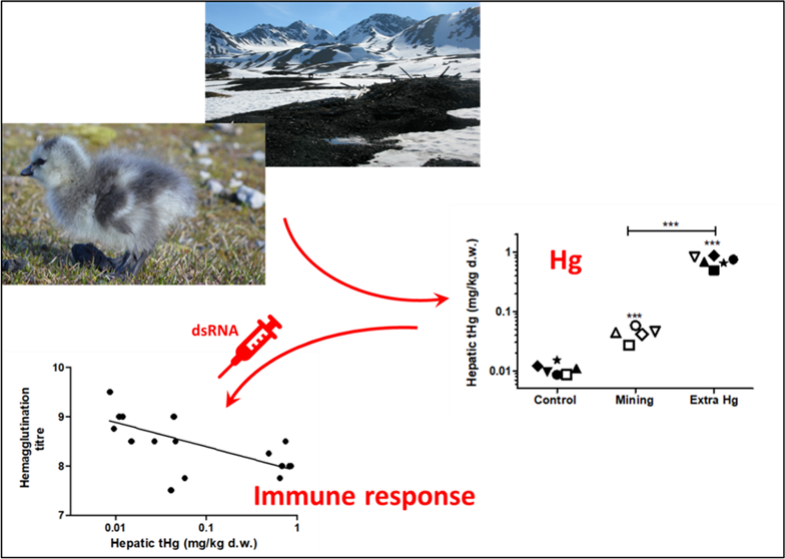

Historical mining activities in Svalbard (79°N/12°E) have caused local mercury (Hg) contamination. To address the potential immunomodulatory effects of environmental Hg on Arctic organisms, we collected newborn barnacle goslings (*Branta leucopsis*) and herded them in either a control or mining site, differing in Hg levels. An additional group at the mining site was exposed to extra inorganic Hg(II) via supplementary feed. Hepatic total Hg concentrations differed significantly between the control (0.011 ± 0.002 mg/kg dw), mine (0.043 ± 0.011 mg/kg dw), and supplementary feed (0.713 ± 0.137 mg/kg dw) gosling groups (average ± standard deviation). Upon immune challenge with double-stranded RNA (dsRNA) injection, endpoints for immune responses and oxidative stress were measured after 24 h. Our results indicated that Hg exposure modulated the immune responses in Arctic barnacle goslings upon a viral-like immune challenge. Increased exposure to both environmental as well as supplemental Hg reduced the level of natural antibodies, suggesting impaired humoral immunity. Hg exposure upregulated the expression of proinflammatory genes in the spleen, including inducible nitric oxide synthase (iNOS) and interleukin 18 (IL18), suggesting Hg-induced inflammatory effects. Exposure to Hg also oxidized glutathione (GSH) to glutathione disulfide (GSSG); however, goslings were capable of maintaining the redox balance by de novo synthesis of GSH. These adverse effects on the immune responses indicated that even exposure to low, environmentally relevant levels of Hg might affect immune competence at the individual level and might even increase the susceptibility of the population to infections.

## Abstract 2

Limited research exists on the immunotoxicity of mercury in wild species, especially in extreme environments like the Arctic. This study reports the immunomodulatory effects of low environmental exposure of Hg in barnacle goslings at a former coal mining site in Svalbard.

## Introduction

Mercury (Hg) is a global contaminant,^1^ potentially affecting the health of both wildlife and human beings. Elevated levels of Hg have been found in remote areas, including the Arctic, mainly due to long-distance transport via air or ocean currents, but also from local emissions.^2,3^ The major sources of local Arctic Hg emissions include coal mining, ferrous and non-ferrous metal industry, and waste incineration.^4,5^ Through the biogeochemical processes in Arctic terrestrial and aquatic environments, Hg can occur in different forms, e.g., elemental (Hg(0)), inorganic forms (mainly Hg(II)), and organic forms (mainly methylmercury, MeHg).^2,6^ Hg (II) is the predominant species in Arctic soil, tundra, snow, and surface ocean,^2,7^ while MeHg is of more concern for high trophic predators (e.g., beluga whales (*Delphinapterus leucas*) and polar bears (*Ursus maritimus*)) comprising more than 80% of their internal total Hg burden, due to its high potential for bioaccumulation and biomagnification.^8,9^

Exposure to trace metals, including Hg, may modulate immune responses in wildlife, even at low environmental levels, and may, as such, potentially result in higher vulnerability to infections. For instance, in studies concerning exposure of birds, Hg was reported to weaken the T-cell-mediated immunity in tree swallows, as assessed by skin swelling responses to phytohaemagglutinin (PHA) challenge,^10^ to decrease macrophage phagocytosis in black-footed albatrosses (*Phoebastria nigripes*),^11^ to lower lipopolysaccharide (LPS) triggered B-cell proliferation in zebra finches (*Taeniopygia guttata*),^12^ and to limit antibody production in common loons (*Gavia immer*).^13^ In addition, parasite loads in female European shags (*Gulosus aristotelis*) were found to be negatively related to the selenium 16-Mar mercury (Se/Hg) molar ratio.^14^ Immunomodulatory effects by trace metals could be either suppressive or stimulatory,^15^ both potentially leading to disordered immune responses. Immune suppression may result in a lowered defense against pathogens, potentially posing higher risks of infection in the individuals and may lead to outbreaks of diseases in the communities.^16,17^ On the other hand, undesired stimulation of the immunity can be costly, especially for migratory birds, which need their energy, e.g., for long-distance flying,^18^ like geese migrating to high-arctic breeding grounds.

Svalbard, Norway (79°N/12°E) is a high-arctic archipelago, where coal mining activities were developed since 1906.^19^ However, because of several fatal accidents and declines in global coal markets,^20^ mines were gradually abandoned and closed but remains were left behind. Historical mining activity and waste piles of abandoned mines in Svalbard have resulted in continued local contamination of soil and vegetation with trace metals, including Hg.^19,21−23^ Although there are several studies illustrating the general health effects of mining-related heavy metal contamination on wildlife, such as Arctic hares (*Lepus arcticus*),^24^ pied flycatchers (*Ficedula hypoleuca*),^25^ voles (*Clethrionmys ritilus* and *Microtus oeconomus*), and small birds (*Carduelis flammea*, *Passerculus sandwichensis*, *Spizella arborea*),^26^ little is known about the trace-metal-induced immunotoxicity in Arctic migratory species.

In an earlier study, an experiment was conducted in 2014 with barnacle goslings (*B. leucopsis*) as a model of Arctic migratory species in Ny-Ålesund, Svalbard, to investigate the effects of legacy mercury from historical mining activities and social isolation on baseline immunity, neurological responses, and stress behavior.^22,27,28^ Environmental Hg exposure by herding goslings in the historical mining area did not affect their baseline immunity, i.e., in nonchallenged birds.^27^ However, to evaluate the overall effects of Hg exposure on the immune system, not only the baseline immunity but also the immune responses upon challenge should be evaluated. To assess this, the current study was designed in which goslings were exposed to environmental Hg, similarly to the earlier study,^27^ and their immune system was challenged with a viral-like stimulus.^29^ In the earlier study, hepatic Hg levels in goslings were relatively low, namely, 0.022 mg/kg dry weight (d.w.) for control goslings and 0.030 mg/kg d.w. for goslings herded in mining areas;^22^ hence, in the present study, an additional group of goslings in the mining area was exposed to extra Hg (as HgCl_2_) via supplementary feed, while the other groups received clean supplementary feed without Hg. The concentrations of the additional Hg in the feed were chosen to be similar to concentrations in the soil from the mining site to reflect realistic exposure to grit intake.^22^ The group receiving supplementary Hg reflected the situation of goslings continuously feeding in the mining area. To minimize the potential effects of genetic variations and maternal Hg exposure, three siblings from each nest were randomly distributed over the three treatment groups. We challenged their immune system 24 h prior to the termination of the experiment through injection of double-stranded RNA (dsRNA) mimicking viral infection.^29^ This viral-like immune challenge was expected to trigger innate immune responses such as inflammation (e.g., expression of proinflammatory cytokines and production of nitric oxide), antiviral response (e.g., expression of antiviral interferon-α, IFN-α), and humoral innate response (e.g., production of nonspecific natural antibodies). After 20 days of exposure, we collected blood and tissue samples to analyze the immune responses.

## Materials and Methods

### Study Site and Animals

**Figure 1:**
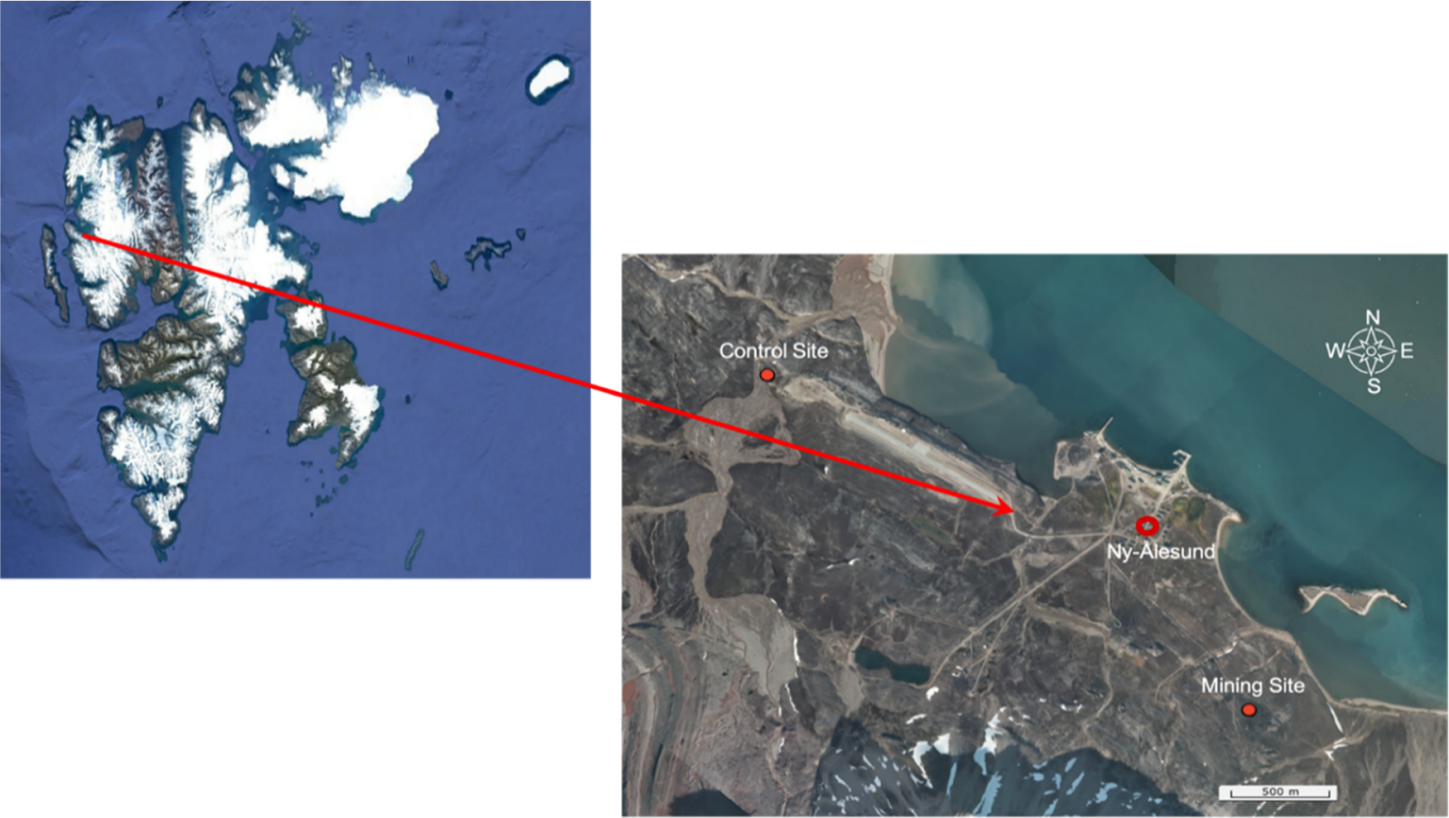
Map showing Svalbard (upper left, source: Landsat/Copernicus) and zoom toward the study sites, including the control site to the northwest and the mining site to the southeast of the village of Ny-Ålesund. The map of the Ny-Ålesund area was adapted from Norwegian Polar Institute via https://toposvalbard.npolar.no/ (Copyright Norwegian Polar Institute).

The study was conducted in the area nearby Ny-Ålesund (78°55′N, 11°56′E), Svalbard (Spitsbergen).^30^ A control site (78°55′54″N, 11°50′10′′E, 2.13 km to the northwest of the Ny-Ålesund village) and a mining site (78°54′55′′N, 11°57′22′′E, 1.36 km to the southeast of the Ny-Ålesund village) were chosen for herding the goslings (Figure 1). The control site is an undisturbed tundra area where wild geese and goslings were also noticed to be grazing (pers. obs.). The mining site experienced historical coal mining activities from 1916 to 1962 and has been abandoned since 1963 due to a severe incident.^31^ Although the vegetation has recovered to some extent during the past decades, remains of the mine activities such as stacks of coal, rusted installations, and abandoned equipment stayed as heritage (pers. obs.). A previous study indicated that both soils and vegetation from the mining site contained significantly higher Hg levels than the control site due to coal mining activities.^22^

Eighteen 0-day-old barnacle goslings were collected from the uncontaminated island Storholmen in Kongsfjorden on 26th June 2019. Three hatchlings were collected per nest from six nests. Immediately upon collection, goslings were labeled with web tags on one of their feet and specific-colored bands on their legs for easy identification during the experiment. Siblings from the same nest (further referred to as siblings) were randomly assigned to three treatment groups, namely, control, mining, and extra Hg group (six goslings per group). Goslings were hand-reared by four humans (BH, NvdB, HvdB, AN) as foster parents, who also trained the goslings for further experimental handling. To minimize the potential effects of parenting by specific individuals, human foster parents took turns to provide care for the different groups of goslings. Body mass and total tarsus length were measured every other day to monitor the development of goslings. One gosling from the mining group was attacked by an arctic fox and dropped out from the experiment on day 13 (9th July 2019).

### Experimental Design

To investigate the effects of environmental Hg exposure on the immune response of barnacle goslings, the three groups of goslings received different treatments for 20 days.^30^ Goslings in the control group were herded into the control site and provided with clean supplementary feed. The additional feed was needed because earlier experiences with herding goslings showed that they could not acquire the resources needed while being herded by humans. Goslings in the mining group were herded in the mining site and given clean supplementary feed, while goslings in the “extra Hg” group were herded in the mining site and exposed to supplementary feed spiked with inorganic Hg (0.14 mg Hg/kg) feed (d.w.) such as HgCl_2_. The concentration of Hg in the feed was selected in the range of concentrations in the soil and vegetation of the mining site.^22^ The two groups in the mining site were herded together in a larger group and were only separated during feeding with the additional feeds (clean versus spiked). The Barnacle goslings in the experiment were feeding on vegetation in either the control or mining site, in which inorganic Hg was the predominant form of Hg.^7^ Furthermore, a relevant source of exposure may also be via ingestion of contaminated soil/coal particles^22^ in which we expected also inorganic Hg to be the main form. Ingestion of grit is common in geese to facilitate the grinding of food in the stomach and can as such be an important source of mercury uptake in our experiment. Therefore, HgCl_2_ was added to the supplementary feed for the extra Hg group (Research Diet Services BV, Wijk bij Duurstede, the Netherlands). Feed was provided from the first day. From day 5 to day 11 (1st to 7th July), goslings were herded in assigned locations for approximately 6 h per day. From day 12 onward, goslings from each group were left overnight in the assigned locations in a cage of approximately 2 m × 2 m × 0.8 m, surrounded by an electric fence to keep out predators. Shelters with electric car seat heaters were installed to keep the goslings warm, and supplemental feed as well as water was provided ad libitum overnight. One day prior to termination of the experiment (day 19, 15th July), one drop of blood was drawn from each gosling by puncturing the brachial vein with a 23G needle (BD Vacutainer, Becton Dickinson) to make a blood smear. The blood smear was air-dried. Then, goslings were immune-challenged with 50 μg/kg body weight poly I:C (synthetic dsRNA analogue) via intraperitoneal (i.p.) injection. This dose was selected based on a study on ducks (*Anas platyrhynchos*) in which they used 100 μg/kg body weight in adult ducks.^32^ Because we studied young goslings, we lowered the challenge concentration by 50% to 50 μg/kg body weight.

During the night of day 19, limited feed was provided to ensure their crop would be empty for euthanasia. We were interested in the different responses to a virus-like challenge based on exposure to mercury, so all goslings were immune-challenged with dsRNA, without testing a nonchallenged subgroup due to the limited sample size.

On day 20 (16th July), goslings were euthanized through decapitation followed by immediate dissection. The duration between immune challenge and euthanasia ranged from 22 to 30 h, and the rank of challenge time was included in the linear regression models for nitric oxide in plasma samples. At least 3 mL of blood per gosling was collected in blood collection tubes coated with K_2_EDTA (BD Vacutainer, Becton Dickinson). Another (air-dried) blood smear was made for each gosling and the rest of the blood was centrifuged at 1000*g* for 10 min to separate plasma and cellular factions. Red blood cells were washed with saline solution (0.9% NaCl) three times. Afterward, plasma and washed red blood cells were snap-frozen in liquid nitrogen and stored at −80 °C until further analyses. Immune organs including the spleen, thymus, and bursa were isolated, snap-frozen, and stored at −80 °C until further analyses for gene expression. Liver tissue was collected and stored at −20 °C to determine the internal Hg and Se levels. All samples were transported to the Netherlands on dry ice.

### Chemical Analyses

Hg and Se levels were determined in both kinds of supplemental feed and in liver tissues. Briefly, liver tissues and feed were freeze-dried at −50 °C for 18 h and then digested in either 70% nitric acid (for Hg analyses) or aqua-regia (for Se analyses) assisted with microwave destruction. The levels of total Hg (*t*Hg) were measured with cold vapor atomic fluorescence spectrometry (CV-AFS),^22,33^ while the levels of Se were measured with inductively coupled plasma mass spectrometry (ICP-MS).^34^ Mussel tissue (ERM-CE278k, European Reference Materials, ERM, Geel, Belgium) and lichen (BCR 482, ERM, Geel, Belgium) were used as reference materials for liver and feed samples, respectively. Blanks were included in each batch of 10 samples. The concentrations of total Hg and Se in liver tissues are expressed as mg/kg dry weight (d.w.).

### Immune Assays

#### Gene Expression

Organs of the spleen, bursa, and thymus were transferred from a −80 °C freezer to a prechilled RNAlater-ICE frozen tissue transition solution (Invitrogen, Breda, the Netherlands) to avoid RNA degradation during handling. Frozen tissues were soaked in at least 10 volumes of the RNAlater-ICE solution overnight before processing to RNA extraction. RNA was extracted from around 15 mg of preserved organs with an RNeasy Mini Kit (Qiagen, Venlo, the Netherlands). The purity and quantity of RNA were checked with a Nanodrop (ND-1000, Themo Scientific, Delaware). Afterward, a QuantiNova Reverse Transcription Kit (Qiagen, Venlo, the Netherlands) was used for reverse transcription reactions to synthesize cDNA with 300 ng RNA input. Gene expression of immune functional genes was assessed with a QuantiNova SYBR Green PCR Kit (Qiagen, Venlo, the Netherlands) on a Rotor-Gene 6000 cycler (Qiagen, Venlo, the Netherlands). A sequence of primer pairs, including the housekeeping gene (GAPDH), immune receptors (CD4, CD8a, MDA5, MHCIa, MHCIIa, RIGI, TLR3, and TLR7), and also immune messengers (IFN-α, IFN-γ, IL8, IL18, iNOS; Biolegio, Nijmegen, the Netherlands) are listed in Table S1.^35,36^ Results were normalized against the housekeeping gene GAPDH and expressed as log 2 fold changes relative to the average of the spleen in the control group by the −ΔΔCT method.^37^ To visualize the results, a heatmap was generated with the online tool Heatmapper.^38^

#### Nitric Oxide Assay

Nitric oxide acts not only as an effector molecule defending the host against pathogens but also as a messenger regulating the immune responses.^39,40^ Therefore, nitric oxide levels can be a good indicator to evaluate the effects of Hg exposure on the immune response upon dsRNA challenge. Nitric oxide levels in gosling plasma samples were measured as described in previous studies.^27,41^ Nitric oxide is not stable in the biological tissues and could be transformed into nitrite (NO_2_^–^) within seconds and then to nitrate (NO_3_^–^) within hours.^41^ Thus, nitrate (NO_3_^–^) was reduced to nitrite (NO_2_^–^), and nitrite (NO_2_^–^) concentrations were measured to represent the nitric oxide levels in the plasma samples. In short, 20 μL of plasma was deproteinized in an alkaline condition, by adding 80 μL of a 75 mmol/L ZnSO_4_ solution and 100 μL of a 55 mmol/L NaOH solution. Then, the mixture was centrifuged for 10 min at 16,000*g*, and 80 μL of the supernatant was transferred to a new tube and mixed with 80 μL of glycine buffer (0.2 mol/L, pH 9.7). Thereafter, nitrate in the samples was reduced to nitrite by cadmium granules coated with copper. Finally, the Griess reaction was used to measure nitrite (μM) in the mixture with a standard curve of NaNO_2_. Nitric oxide levels in plasma (μM) were calculated as 20× nitrite in the mixture.

#### Hemolysis–Hemagglutination Assay

Natural antibodies (NAbs) and complement contribute to the first immune defense in animals without any infection history.^42,43^ As NAbs are nonspecific, they can neutralize various pathogens, resulting in agglutination. Together with the lysis of pathogens via the complement pathway, a broad range of pathogens can be inhibited and eliminated.^44^ A hemolysis–hemagglutination assay was used to evaluate the interaction of NAbs and complement (with lysis titers) and for NAbs activity (with agglutination titers).^27,45^ Briefly, gosling plasma was serially two-fold diluted 10 times in round (U) bottom 96-well plates with phosphate-buffered saline (PBS, Gibco, Paisley, U.K.). Afterward, the same volumes of diluted plasma and 1% rabbit blood cell suspension in PBS were mixed and incubated in a 37 °C humidified incubator for 90 min. Then, the plates were tilted to a 45° angle on the long axis at room temperature for 30 min to enhance visualization for scoring hemagglutination titers. After incubation at room temperature for another 70 min, hemolysis titers were determined. All of the samples were observed and visually scored^44^ by one person (BH). Half scores were given when agglutination or lysis was intermediate. Two replicates were tested for each gosling.

#### Haptoglobin Assay

Haptoglobin is an acute-phase protein rapidly increasing in the case of inflammation, infection, or trauma.^46−48^ A commercially available colorimetric haptoglobin assay kit (TP801; Tri-Delta Development Limited, Maynooth, Ireland) was used to quantify the haptoglobin-like activity (mg/mL) in gosling plasma samples with a calibration curve. As hemolysis interfered with the assay, plasma sample redness was measured as absorbance at 450 nm before the addition of chromogen reagent for statistical correction.^47^

#### Blood Smear

Blood smears were stained with Hemacolor Rapid staining (Sigma-Aldrich, Zwijndrecht, the Netherlands) and counted by one observer (BH) using a light microscope at 1000× magnification with immersion oil (Zeiss, Jena, Germany). To determine the leukocyte density, the number of leukocytes, thrombocytes, and red blood cells was counted until the vision contained the 5000th red blood cell (usually 50–60 visions). For leukocyte counts, heterophils (normal or toxic), eosinophils, basophils, monocytes, and lymphocytes (reactive or nonreactive) were identified according to the morphological characteristics,^49,50^ and at least 100 leucocytes were counted per slide. Changes (Δ) in leucocyte populations were calculated by subtracting the results of the slide before poly I:C challenge from the one after the challenge.

### Oxidative Stress Assays

Indicators for oxidative stress were measured in gosling red blood cells or in plasma, including superoxide dismutase (SOD), glutathione peroxidase (GPx), total glutathione (GSHt), glutathione disulfide (GSSG, oxidized form of glutathione), and malondialdehyde (MDA) in red blood cells (RBCs), together with retinol (vitamin A), lutein (carotenoids), α-tocopherol (vitamin E), and MDA in plasma, according to the methods documented in previous studies.^51,52^ Briefly, an automated spectrophotometer A25-Autoanalyzer (BioSystems S.A., Barcelona, Spain) was used to quantify the SOD, GPx, GSHt, and GSSG in RBCs. A high-performance liquid chromatography (HPLC) system was used to measure the MDA in both RBCs and plasma samples as well as retinol, lutein, and α-tocopherol in plasma samples.

### Genetic Sexing

The sex of goslings was determined genetically (Bird Genetics, Erp, the Netherlands) with DNA extracted from erythrocytes after the field experiment. The CHD-Z and CHD-W gene fragments located on the avian sex chromosome (either Z or W) were checked by the polymerase chain reaction (PCR).^53^ After separating PCR products on agarose gel, one band refers to a male (only the CHD-Z gene on two Z chromosomes) and two bands refer to a female (both CHD-Z and CHD-W genes on Z and W chromosomes). Sexing results showed that we had 7 males and 10 females in total. In both control and extra Hg groups, there were one male and five females, while all of the five goslings in the mining group were male.

### Statistics

All statistical analyses were performed with SPSS (IBM SPSS Statistics, version 25). First, we checked whether the growth of goslings was affected by Hg exposure during the experiment, using linear regression models. Final body mass (g) or total tarsus length (mm) measured on day 19 were used as dependent variables, while log hepatic *t*Hg, sex, nest, and body mass or tarsus length on day 1 were set as independent variables.

Endpoints were analyzed with either ordinal regression (for discrete data, e.g., hemolysis and hemagglutination titers) or linear regression (for continuous, e.g., gene expression, blood cell population, GSHt, etc.) models. Variables were transformed if they did not meet the model assumption of normal distribution. For instance, internal Hg and Se levels and the heterophil/lymphocyte (H/L) ratio were log-transformed, while haptoglobin and nitric oxide levels were square-root-transformed.^27^ Hepatic Hg levels and sex were included in the models as independent variables. To account for parental effects (“nest”), the nest number was included as a random variable in the regressions. For nitric oxide, the ranking of challenge time was also included in the linear regression model, while plasma redness (absorbance at 450 nm) was added in the model for haptoglobin as a covariate.^48^ Results were visualized with GraphPad Prism 5 (San Diego, CA). A level of α = 0.05 was used as the threshold for the statistical significance of analyses.

## Results and Discussion

The study aimed to explore the effects of environmental Hg exposure on immune responses upon a viral-like challenge in barnacle goslings. We assessed multiple immune functional endpoints, including changes in the immune cell population, plasma-based immune indicators (hemolysis, hemagglutination, haptoglobin, and nitric oxide), immune gene expression, and oxidative stress. With the challenge of the immune system with dsRNA and increased Hg exposure, more specific effects were quantified than in a previous study on nonchallenged barnacle goslings at the same location.^27^

### Effects of Exposure on Growth

According to the output of linear regression models, the final body mass or tarsus length on day 19 was not influenced by hepatic *t*Hg levels or the measurements on day 1 (Table 1). However, “nest” showed significant effects on the final body mass, indicating a parental effect, while the tarsus length of males (74.0 ± 1.4 mm (average ± stdev), *n* = 7) was significantly longer than that of females (69.2 ± 0.8 mm, *n* = 10; Table 1). No significant differences were detected in the last measurement on day 19 for body mass (g) among different treatment groups (control group: 484.0 ± 70.2 g; mining group: 515.6 ± 70.5 g; extra Hg group: 478.7 ± 45.4 g) or tarsus length (mm, control group: 70.7 ± 2.5 mm; mining group: 73.1 ± 3.6 mm; extra Hg group: 70.2 ± 4.4 g). The lack of effects of exposure on the growth rate is likely because the Hg exposure is low and ad libitum feed was available for the goslings.

**Table 1:** Statistical Outcomes of Linear Models for Gosling Growth during the Experiment Calculated with Body Mass (g) and Tarsus Length (mm)^a^

	variable	estimate	SE	*t*	*p*
final body mass (g)	log *t*Hg				0.685
	sex	38.251	26.797	1.427	0.179
	nest	22.925	7.251	3.162	0.008
	body mass on day 1	3.891	2.749	1.416	0.182
final tarsus length (mm)	log *t*Hg	0.327	1.060	0.308	0.763
	sex	4.495	1.493	3.011	0.011
	nest	0.405	0.599	0.676	0.512
	tarsus length on day 1	0.730	0.665	1.098	0.294

a Underlined *t*- and *p*-values are significant at *p* < 0.05.

### Hepatic Hg and Se Levels

**Figure 2:**
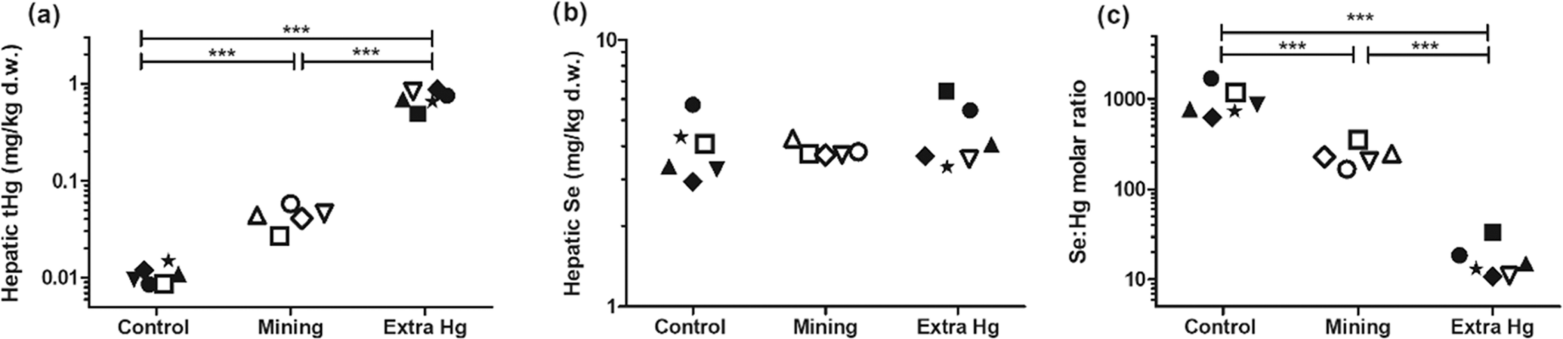
Hepatic *t*Hg levels (a) and Se (b) levels in gosling and the calculated Se/Hg molar ratio (c) of different exposure groups. Siblings from the same nest are shown as symbols with the same shape, while males are represented as closed symbols and females as black symbols. Significant differences were checked with one-way ANOVA with the Tukey post hoc test (***p < 0.001).

Total Hg levels in gosling liver tissues ranged 3 orders of magnitude among goslings (from 0.0086 up to 0.87 mg/kg d.w.) and showed a significant increase from the control group (0.011 ± 0.002 mg/kg d.w.) to the mining group (0.043 ± 0.011 mg/kg d.w.) and to extra Hg group (0.713 ± 0.137 mg/kg d.w., Figure 2a). According to the linear regression, liver Hg levels were independent of the Se level, sex, and “nest” (Table 2). Compared to the previous study,^22^ the *t*Hg levels in the control group were lower (mean current study: 0.011 mg/kg d.w. vs mean previous study: 0.022 mg/kg d.w.), while those in the mining group were higher (mean current study: 0.043 mg/kg d.w. vs mean previous study 0.030 mg/kg d.w.). The reason for these differences could be related to the different herding styles. In the previous study, goslings were walked all the way from the village of Ny-Ålesund to either the control or mining sites. Hence, the control goslings could be exposed to more Hg than in the control site while walking. In the current study, goslings were transported by the foster parents, which ensured that the goslings were only exposed to local Hg in either the control or mining sites. The higher *t*Hg levels in the mining group of the current study were probably because of the longer grazing time as the goslings were left in the sites overnight from day 12 onward. The extra Hg group was provided with supplementary feed with 0.14 mg Hg/kg feed d.w., which was at the same level as Hg levels found in the soil of the mining site.^22^

**Table 2:** Output of Ordinal (for) or Linear Regression Models for Hepatic Element Levels, Immune Response, and Oxidative Stress Endpoints^a^

	endpoints	log *t*Hg (*t*/wald)	log Se (*t*)	sex (*t*/wald)	nest (*t*/wald)	challenge time order	total df	regression (*F*/χ2)
hepatic levels	log *t*Hg (mg/kg d.w.)		1.005	–0.490	0.729		16	0.435
	log Se (mg/kg d.w.)	1.005		–0.034	**–3.539**		16	**4.751**
	Se/Hg molar ratio	**–6.401**	1.353	–1.652	0.172		16	**10.897**
immune response	hemolysis	1.759		**7.003**	3.050		93	**9.307**
	hemagglutination	**8.071**		0.613	0.173		109	**10.536**
	haptoglobin-like activity	–0.028		0.373	**–3.656**		16	**4.501**
	Δheterophils (%)	**2.257**		–2.015	0.458		16	**3.555**
	Δlymphocytes (%)	–0.495		**2.337**	1.298		16	3.152
	Δlog H/L	1.516		–2.054	–0.679		16	2.953
	log 2 IL18 expression	**2.221**		–1.894	–0.462		16	**3.574**
	log 2 iNOS expression	**2.555**		–0.150	0.879		16	2.496^b^
	nitric oxide (μM)	–1.744		–0.234	0.427	**2.449**	16	**4.157**
oxidative stress	GSHt (μmol/g RBC)	**3.002**		0.459	0.356		16	**3.062**
	GSHox (μmol/g RBC)	1.884		–0.773	–0.437		16	1.575
	GSHox%	0.197		–1.045	0.165		16	0.405
	tocopherol (nmol/mL plasma)	–1.501		–0.233	**–2.255**		16	2.559
	retinol (nmol/mL plasma)	0.129		–0.055	**–2.506**		16	2.212

a Listed are the χ2 (for hemolysis and hemagglutination) or *F*-values of overall regression and wald (for hemolysis and hemagglutination) or *t*-values of individual parameters. Underlined *t*- and *F*-values are significant at *p* < 0.05. N (control/mining/extra Hg) = 5/6/5.

b The overall regression for iNOS expression is not significant when sex and “nest” are included (*F*-value = 2.496, *p*-value = 0.106). When sex and “nest” are excluded, the linear regression is significant (*F*-value = 7.310, *p*-value = 0.016) due to a lower degree of freedom (df).

Se has been documented to be protective against Hg toxicity.^54−57^ Although Se levels in liver tissues did not vary among exposure groups (Figure 2b), Se levels differed significantly between “nests” (Table 2), suggesting potential maternal influence.^58^ We also calculated the Se/Hg molar ratios, ranging approximately from 1000 to 10, which were significantly different among exposure groups (Figure 2c). The Se/Hg molar ratio can be used as an indicator to estimate Hg toxicity, as Se showed a protective effect on Hg intoxication.^14,57^ In an earlier study, the number of parasites related negatively to Se/Hg molar ratios in a range from 5 to 30 in female shags.^14^ This range partly overlapped with the Se/Hg ratios we found in the goslings (10–1000). In our results, the hepatic Se levels did not differ among goslings and therefore did not influence the differences in Se/Hg molar ratios among the groups (Table 2). Therefore, the hepatic Se level was not included as one of the independent factors in the linear models for immune and oxidative stress makers.

### Immune Responses

#### Gene Expression

**Figure 3:**
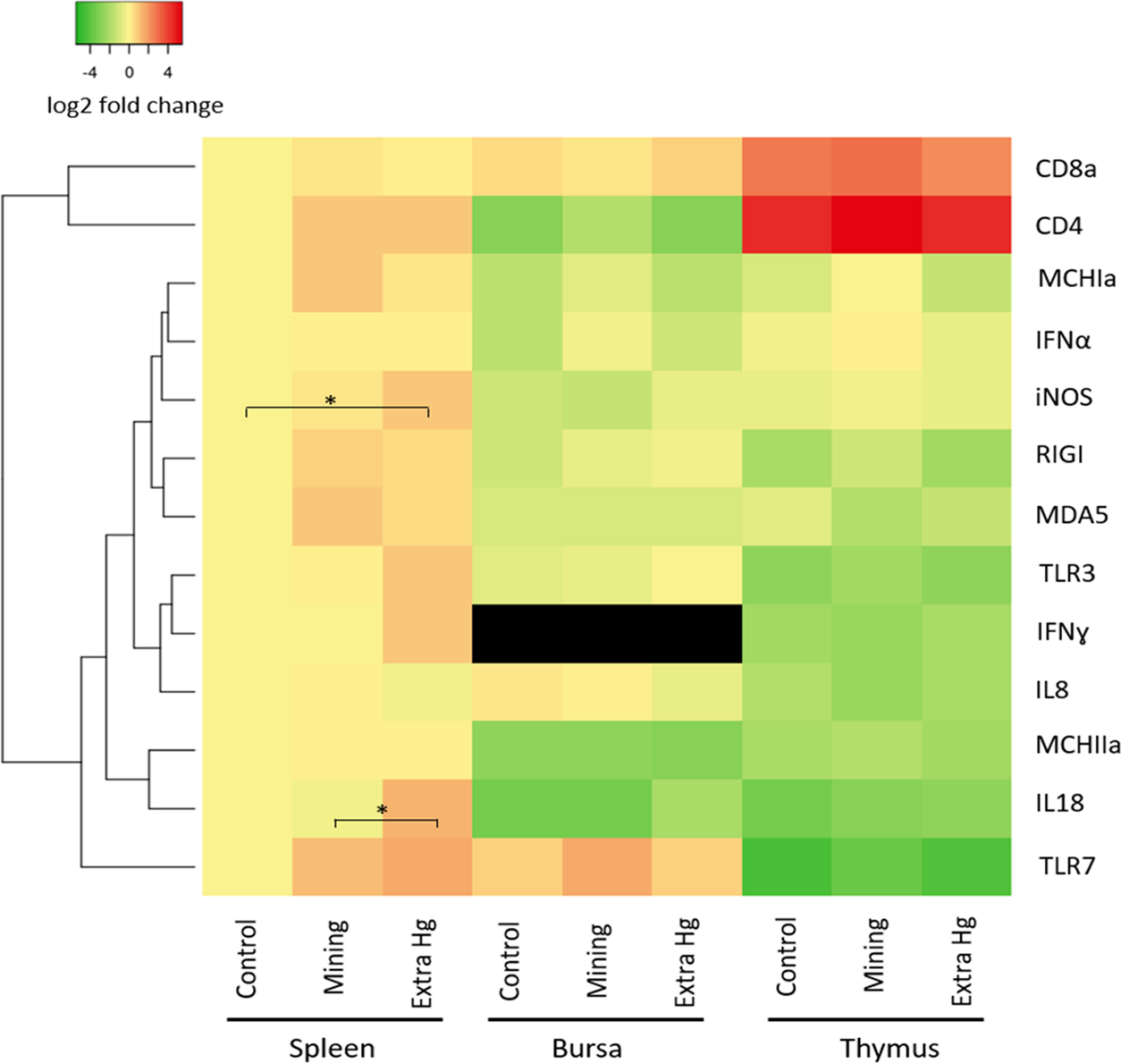
Gene expression of immune functional genes in the gosling spleen, bursa, and thymus tissues. Results were normalized first with the housekeeping gene GAPDH and shown as the log 2 fold change relative to the average expression in the spleen of the control group. The black color indicated the nondetected genes (Ct > 40, IFN-γ in the bursa). Significant differences between groups (*n* = 6 for the control and extra Hg group, and *n* = 5 for the mining group) were tested with one-way ANOVA for each gene per organ with the Tukey post hoc test (*p < 0.05).

Gene expression profiles were integrated into a heatmap (Figure 3). Different patterns of gene expression were shown in the three organs we tested. For example, T-lymphocyte-specific receptors, including the cluster of differentiation 4 (CD4) and CD8 receptors were much higher expressed in the thymus than in the spleen and bursa, while toll-like receptor 7 (TLR7) was lower expressed in the thymus (Figure 3). These variable gene expression profiles in different organs are due to the different compositions of immune cell populations in the organs, namely, T- and B-lymphocytes are the major cell types in the thymus and bursa, respectively, while the spleen contains multiple types of immune cells.^59^

Among all of the tested genes, only the expression of iNOS and IL18 in the spleen was significantly different among exposure groups. The expression of iNOS in the extra Hg group was significantly higher than that in the control group, while the expression of IL18 was upregulated in the extra Hg group compared to the mining group. According to the significant linear regression models, the expression of spleen iNOS and IL18 were positively correlated with the internal *t*Hg levels (Table 2). As both iNOS and IL18 are proinflammatory genes,^60−62^ the upregulation suggests a potential increased inflammation due to Hg exposure upon challenge. The undesired inflammation might result in disorders in immunity such as autoimmunity^63^ and could be costly for birds, especially for the ones who need energy for migration.^64^

#### Nitric Oxide

**Figure 4:**
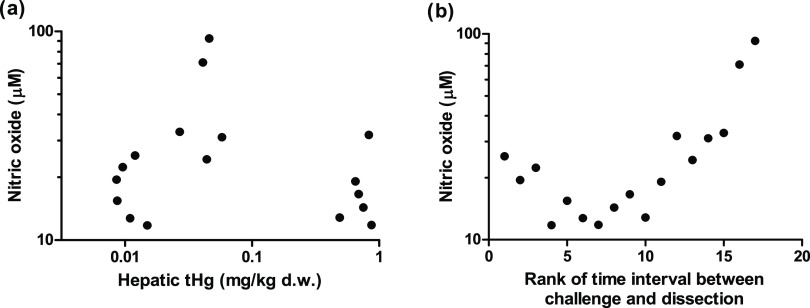
Nitric oxide levels in the gosling plasma against the hepatic *t*Hg levels (a) and the rank of the time interval between challenge and dissection (b).

Plasma nitric oxide levels were not significantly affected by Hg exposure but were strongly related to the time interval between challenge and dissection (Table 2 and Figure 4). The highest nitric oxide levels (up to 92.56 μM) were found in the goslings with the longest challenge time (around 30 h from the injection of poly I:C to dissection). Nitric oxide levels after the dsRNA challenge were higher (control group: 17.87 ± 4.99 μM; mining group: 50.40 ± 26.65 μM; extra Hg group: 17.76 ± 6.77 μM) than the baseline nitric oxide levels measured by a previous study^27^ on barnacle goslings in the same area (mean control group: 0.69 μM; mean mining group: 0.35 μM). The nitric oxide levels were related to the timing between the challenge and measurement of the levels (Figure 4b), which would indicate that challenge time probably should have been longer to ensure a full induction of nitric oxide production. Barnacle goslings are waterfowl, and Pekin ducks (*Anas platyrhynchos domesticus*) only showed significantly higher levels of nitric oxide in serum after 72 h post-infection with H5N1 avian influenza virus, while chickens already produced significantly higher nitric oxide 24 h post-infection.^65^ Hence, it may be that waterfowl species need a longer challenge time to build a proper nitric oxide response in comparison to the commonly studied chicken. Nevertheless, Hg exposure upregulated the gene expression of iNOS at the transcriptional level in the goslings (Figure 3), which is a relatively early-stage indicator of a nitric oxide response. Therefore, the nonchanged plasma nitric oxide levels are not in conflict with the upregulated iNOS expression.

#### Hemolysis–Hemagglutination

The hemolysis titer was not significantly influenced by the hepatic *t*Hg levels but was significantly higher in males than in females (Table 2). The hemolysis titer reflects the activity of complement-like enzymes^66^ and was reported to be male-biased in adult free-living wild birds during the breeding season.^67^ Male Barnacle goslings also showed significantly higher hemolysis titers (*p*-value = 0.024) in the previous study.^27^ Due to the relatively small sample size we used, all of the five goslings in the mining group were male, showing significantly higher hemolysis titers than the other two groups. Thus, although the goslings we used in the current study were at an early stage of development and much younger than the age for sexual maturity (2 years of age),^68,69^ our results demonstrated that sex instead of Hg exposure was the major driver of the differences in the hemolysis titer.

**Figure 5:**
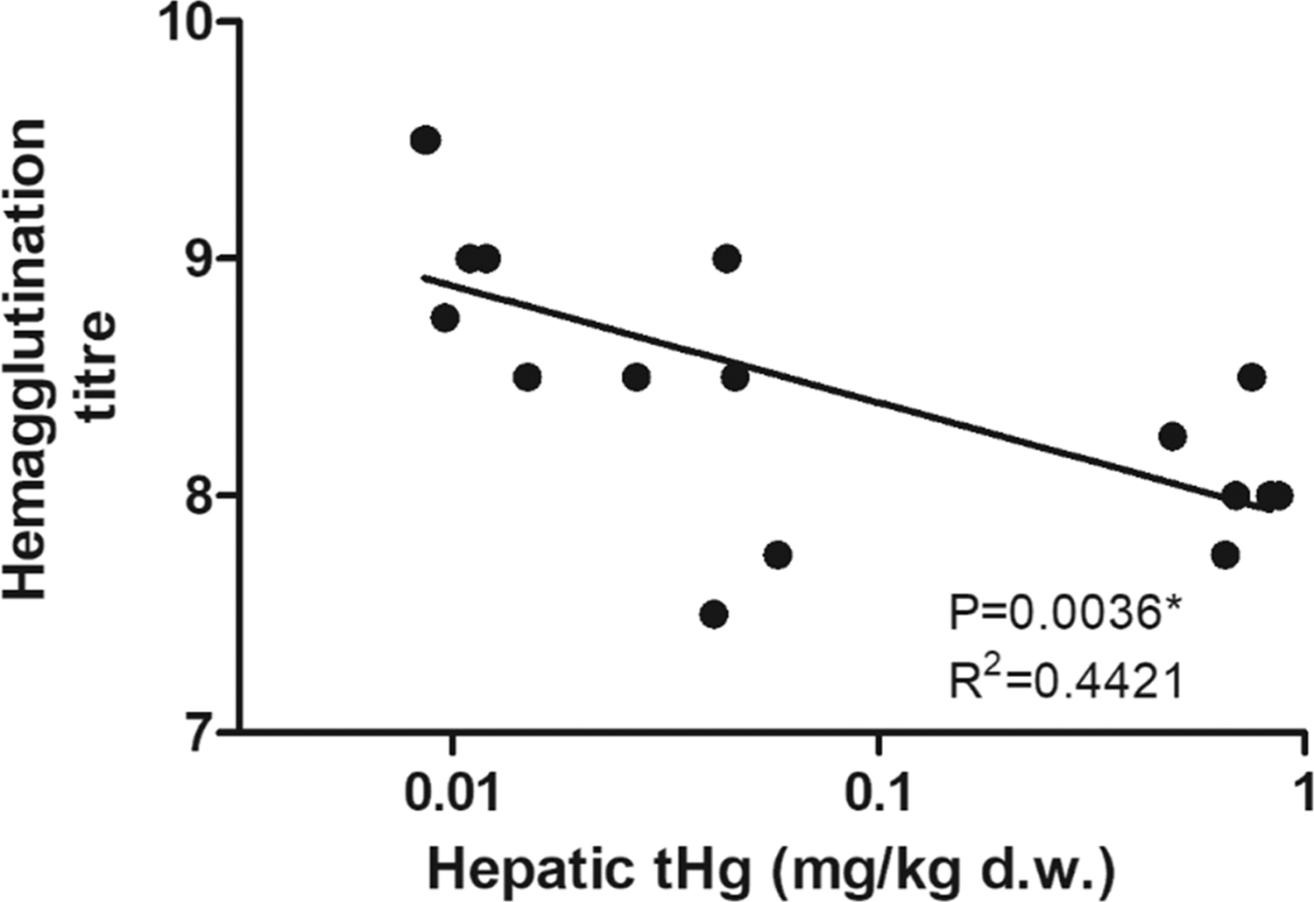
Linear regression of hemagglutination titer against *t*Hg levels in gosling liver samples (for statistical details, see Table 2).

As for the hemagglutination titer, hepatic *t*Hg levels showed a significant negative influence according to the linear regression model (Table 2 and Figure 5). Natural antibodies involved in the hemagglutination process are important for the constitutive innate immunity, providing the rapid first line of defense to antigens,^45^ and are also crucial players in the humoral immunity mediated by B-lymphocytes.^70^ In the previous study, natural antibody activity showed a decrease in goslings herded in the mining site only after social isolation, which acted as an acute stressor.^27^ Higher natural antibody levels were predictive of higher survival rates in laying hens.^71^ Besides, natural antibodies were reported to protect mice from viral and bacterial infections by suppressing pathogen spreading and enhancing pathogen elimination in lymphoid organs.^72^ Therefore, in the current study, the significantly lower natural antibody activity due to Hg exposure suggests an impaired constitutive innate humoral immunity and defense against pathogens, which might lead to a higher risk of infections for the goslings.

#### Haptoglobin

Haptoglobin-like activity in plasma samples showed no difference among the exposure groups (control group: 0.335 ± 0.075 mg/mL, mining group: 0.346 ± 0.072 mg/mL, and extra Hg group: 0.335 ± 0.062 mg/mL). Linear regression also indicated that haptoglobin levels were not influenced by internal *t*Hg or sex, although they differed between “nests” (Table 2). Plasma redness (as absorbance at 450 nm) was also included in the linear regression model but had no effect (*t*-value = −0.557, *p*-value = 0.589).

#### Immune Cell Populations

**Figure 6:**
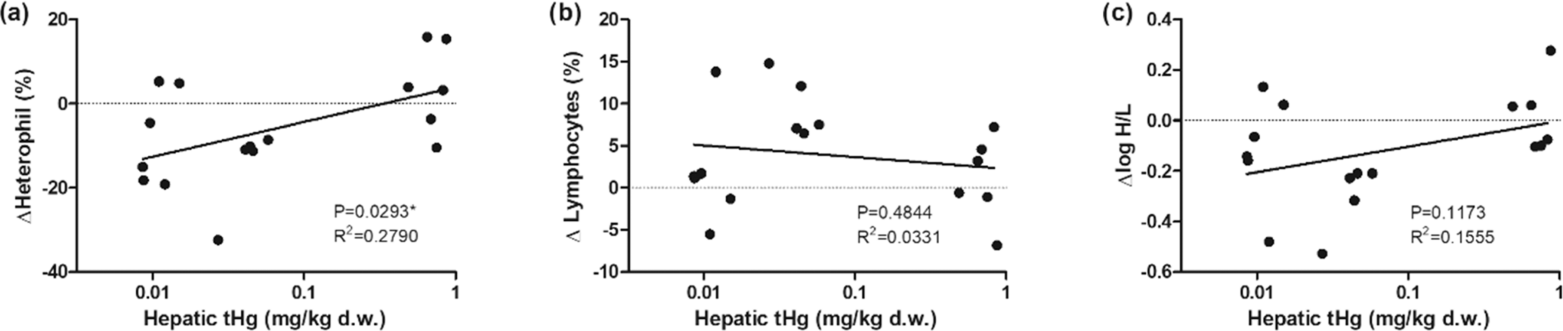
Change of heterophil population (a), lymphocyte population (b), and log heterophil/lymphocyte ratio (c) in relation to *t*Hg levels in liver samples according to the blood smear readings (for statistical details see Table 2).

Immune cell populations were assessed using blood smears and the percentage of each type of leukocytes within the total white blood cells (WBCs) was calculated. The change (Δ) of each cell type of leukocyte due to the immune challenge (expressed as a percentage in relation to total WBCs) was calculated as a proxy for the immune responses. ΔHeterophils showed a significant positive correlation with hepatic *t*Hg levels (Table 2 and Figure 6a). ΔLymphocytes values were slightly decreased with the increase of hepatic *t*Hg levels (not significant) and were found to be significantly influenced by sex (Table 2and Figure 6b). With the increase of Δheterophils and decrease of Δlymphocytes in the extra Hg group, the Δlog heterophil/lymphocyte ratio (Δlog H/L) increased with the higher hepatic *t*Hg levels, although not significant (*p*-value = 0.153) and was independent of all of the factors in the model (Table 2 and Figure 6c).

Heterophils in avian species have a similar function as neutrophils in mammals, being phagocytic cells, protect the organisms against pathogens, and are also one of the major cell types producing nitric oxide.^73^ Lymphocytes include B-cells that induce the antibody, mediating humoral immunity, T-cells, and cellular immunity.^74^ However, the microscopic method used for cell type identification cannot differentiate B- and T-cells. Nevertheless, the H/L ratio is an indicator of humoral immune response^75−77^ and an increased H/L ratio, although not significant, may be related to lower humoral immunity in exposed goslings. Adverse effects of inorganic Hg on immune cell populations have also been reported in other species. For example, abnormally high levels of heterophils were found in American kestrels (*Falco sparverius*) exposed to Hg,^76^ while reduced CD4+ lymphocyte counts were reported in Hg-exposed workers.^78^ In vitro exposure to HgCl_2_ inhibited the proliferation of immune cell lines from both chicken (*Gallus gallus domesticus*)^79^ and murine species, as well as primary lymphocytes from mice (*Mus musculus*),^80^ beluga whales,^81^ snakes (*Nerodia taxispilota*),^82^ and humans (*Homo sapiens*).^83^ Different sensitivities of immune cell types to Hg exposure may explain the change in immune cell composition.^79^

### Oxidative Stress

**Figure 7:**
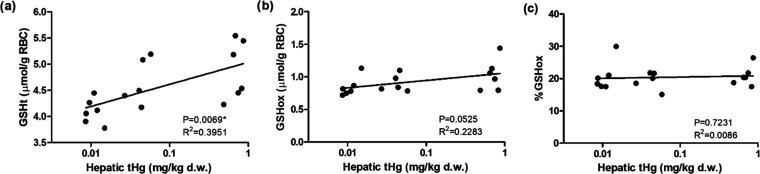
Levels of total glutathione (GSHt, a), oxidized glutathione (GSHox, b), and the percentage of oxidized glutathione (GSHox%, c) in gosling red blood cells (RBCs; for statistical details, see Table 2).

Hg is reported to have a high affinity for the thiol group in GSH^84^ and could induce the conversion of GSH to its oxidized form glutathione disulfide (GSSG), disturbing the redox balance and resulting in oxidative stress.^85−87^ Levels of total GSH (GSHt) and GSSG were qualified in red blood cells. According to the linear regression models, hepatic *t*Hg levels significantly increased GSHt levels (*p*-value = 0.0069) and almost significantly increased GSHox levels (*p*-value = 0.0525) (Table 2 and Figure 7a,b). As two GSH molecules can be oxidized into one GSSG (2GSH + H_2_O_2_ ⇌ GSSG + H_2_O),^88^ the levels of oxidized GSH molecules (GSHox) were calculated by duplicating the levels of GSSG measured (Figure 7b). To evaluate the redox status, the percentage of oxidized GSH molecules (GSHox%) was calculated (GSHox% = GSHox/GSHt; Figure 7c). Due to the increasing trend of both GSHt and GSHox with the increase of internal *t*Hg levels, the GSHox% stayed more or less stable along with the increase of internal *t*Hg levels (Figure 7b,c). Thus, our results demonstrate that goslings probably were able to compensate for the Hg-induced GSH depletion by triggering the de novo synthesis of GSH at the same time as a protective strategy. This dynamic feedback resulted in a stable GSHox% and illustrated that goslings were able to maintain redox balance upon Hg exposure at these environmentally relevant levels. Similarly, increased GSHt and GSHox with stable GSHox% were found in captured mallard ducks with more than 20 μg Pb/dL blood.^52^ Hg and Pb are both divalent metals, and they probably have a similar mode of action for toxicity. However, when the Hg exposure gets higher, potentially exceeding the capacity of the buffer system, the levels of GSH could decrease, resulting in oxidative stress. For example, in surf scoter (*Melanitta perspicillata*), hepatic GSH levels showed a significant negative correlation with hepatic *t*Hg levels ranging from 10 to 30 mg/kg d.w.,^89^ which is around 10 times higher than the highest exposure in our study.

No effect of Hg exposure was found in other indicators for oxidative stress, including SOD, GPx, MDA, retinol, lutein, and α-tocopherol.

### Effects of Nest and Sex

Nest, as an indicator of potential maternal effects, and sex were included in the statistics (Tables 1 and 2). ‘Nest’ influenced the internal Se levels, probably reflecting maternal exposure.^58^ Haptoglobin-like activity, tocopherol, and retinal levels were significantly correlated to “nest,” but none of them were affected by internal *t*Hg levels. Thus, the maternal effects on these immune endpoints might be related to maternal exposure to Se or genetic variations between nests.

Sex only showed an influence on hemolysis titers and Δlymphocytes (Table 2). Females are reported to usually have greater immune responses than males, such as phagocytosis and antibody responses.^90^ However, this sex-related difference is probably too early to be expected on most endpoints we measured at such a young age. Besides, due to the small sample size in the study, males and females were not evenly distributed in each group, especially in the mining group, where all five of the goslings turned out to be male. Larger sample size and more insights into the modes of action underlying the sex-dependent immune responses are needed to confirm and explain our findings.

In summary, we revealed that even at low environmentally relevant levels, Hg exposure was related to changes in immune responses upon a viral-like immune challenge in barnacle goslings. The results of the current study, however, may need to be interpreted with some care due to the practical and also ethical constraints of experimental field studies like the current one, especially with respect to the limited number of animals in the experimental groups. Nonetheless, the study indicates that Hg exposure led to a weaker innate humoral immune response with lower levels of natural antibodies and also induced inflammation by upregulating the gene expression of iNOS and IL18. In addition, Hg exposure in our study appeared to oxidize GSH to GSSG, but goslings managed to compensate for this effect and maintained the redox balance by synthesizing more GSH. The observed inflammation due to Hg exposure could be costly for migratory birds like barnacle geese and influence their overall fitness. These adverse effects on the immune response, especially on innate humoral immunity, may result in compromised immune competence with weaker defense against infections. Some issues, however, still need further research, e.g., the effects on the later stage immune response such as nitric oxide levels. Compared with the previous study on baseline immunity, more adverse effects were noticed in the challenged immune responses included in the present study, indicating that in future immunotoxicity studies, attention should be focused on the stimulated immune responses in addition to baseline immunity.

## Supporting Information Available

List of primers used for qPCR (PDF)
