# Supplementary material for: Mercury-Modulated Immune Responses in Arctic Barnacle Goslings (Branta leucopsis) upon a Viral-Like Immune Challenge
Source: Environ Sci Technol. 2023 Mar 20;57(13):5337–48. doi: 10.1021/acs.est.2c07622 (PMC10077589; doi:10.1021/acs.est.2c07622)
Supplement: Supplementary file 1 — es2c07622_si_001.pdf [file es2c07622_si_001.pdf]

Supporting Information for the Manuscript:

Mercury modulated immune responses in Arctic Barnacle goslings (*Branta leucopsis*) upon a viral-like immune challenge

Biyao Han[1], Hans van den Berg[1], Maarten J.J.E. Loonen[2], Rafael Mateo[3], Nico W. van den Brink[1*]

1. Wageningen University, Division of Toxicology, The Netherlands

2. University of Groningen, Arctic Centre, the Netherlands

3. Research Institute of Hunting Resources (IREC - CSIC, UCLM, JCCM), Spain

This SI-file contains:

Table S1. List of primers used for qPCR

Number of Pages: 2 (including cover sheet).

Table S1. List of primers used for qPCR

Gene	Forward Primer 5'-3'	Reverse primer 5'-3'	Reference
GAPDH	CATCTTCCAGGAGCGCGACC	AGACACCGGTGGACTCCACA	He et al 2017
CD8a	AGAGACGAGCAAGGAGAA	GACCAGGGCAATGAGAAG	He et al 2017
CD4	TTTCAACGCCACAGCAGA	GTGCCTCAACTGGATTTT	He et al 2017
IFN-α	CAGCACCACATCCACCAC	TACTTGTTGATGCCGAGGT	He et al 2017
IL8	CTCCTGATTTCCGTGGCTCT	AGCACACCTCTCTGTTGTCC	He et al 2017
IL18	TGAAATCTGGCAGCGGAATGAAC	TCCCATGTTCTTCCTCACAACA	Xu et al 2016
iNOS	GAACAGCCAGCTCATCCGATA	CCCAAGCTCAATGCACAACTT	Xu et al 2016
MDA5	TGCTGTAGTGGAGGATTTG	CTGCTCTGTCCCAGGTTT	He et al 2017
MHCIa	GAGCAAGCAGGGGAAGGA	CCGTTAGACACTGGGGTT	He et al 2017
MHCIIa	CGGCCAGTTCATGTTCGAT	AAGCTGGCAAACTTCGAGA	He et al 2017
RIGI	AGCACCTGACAGCCAAAT	AGTGCGAGTCTGTGGGTT	He et al 2017
TLR3	CAGCAAATTTAGGATGGCAAC	ACAGATTTCCAATTGCACGTA	He et al 2017
TLR7	CACAGAAAAATGGTACCTC	TACATCGCAGGGTAAACT	He et al 2017
